# Rechained: Sybil-Resistant Distributed Identities for the Internet of Things and Mobile Ad Hoc Networks

**DOI:** 10.3390/s21093257

**Published:** 2021-05-08

**Authors:** Arne Bochem, Benjamin Leiding

**Affiliations:** 1 Institute of Computer Science, University of Goettingen, 37077 Goettingen, Germany; 2 Institute for Software and Systems Engineering, Clausthal University of Technology, 38678 Clausthal-Zellerfeld, Germany; benjamin.leiding@tu-clausthal.de

**Keywords:** Internet of Things, Identity, security, authentication, Sybil attack, blockchain, self sovereign identity

## Abstract

Today, increasing Internet of Things devices are deployed, and the field of applications for decentralized, self-organizing networks keeps growing. The growth also makes these systems more attractive to attackers. Sybil attacks are a common issue, especially in decentralized networks and networks that are deployed in scenarios with irregular or unreliable Internet connectivity. The lack of a central authority that can be contacted at any time allows attackers to introduce arbitrary amounts of nodes into the network and manipulate its behavior according to the attacker’s goals, by posing as a majority participant. Depending on the structure of the network, employing Sybil node detection schemes may be difficult, and low powered Internet of Things devices are usually unable to perform impactful amounts of work for proof-of-work based schemes. In this paper, we present Rechained, a scheme that monetarily disincentivizes the creation of Sybil identities for networks that can operate with intermittent or no Internet connectivity. We introduce a new revocation mechanism for identities, tie them into the concepts of self-sovereign identities, and decentralized identifiers. Case-studies are used to discuss upper- and lower-bounds for the costs of Sybil identities and, therefore, the provided security level. Furthermore, we formalize the protocol using Colored Petri Nets to analyze its correctness and suitability. Proof-of-concept implementations are used to evaluate the performance of our scheme on low powered hardware as it might be found in Internet of Things applications.

## 1. Introduction

The persistent growth and expansion of the Internet of Things (IoT) [1,2], the progressing digitization of our daily life [3,4], and the emergence of complex machine-to-machine, or machine-to-human transaction and interaction scenarios [5], results in a growing popularity of wireless ad hoc networks such as mobile ad hoc networks (MANETs) or vehicular ad hoc networks (VANETs). While participants of the Internet of Things should be always connected to the Internet by default, MANETs and their sub-types are often heavily partitioned, with transient connections occurring between nodes due to their mobility, resulting in a constantly changing network topology. Moreover, communication in MANETs is usually organized in a decentralized manner without a connection to any central authority or the Internet [6,7]. However, even IoT scenarios have to account for Internet disconnects and short period of ad hoc organization due to missing coverage or temporary disconnects, e.g., [8,9].

The growing popularity raises the issue of providing proper security mechanisms. Without such, the distributed nature of ad hoc networks and their lack of a central authentication authority leaves them easy targets for Sybil attacks. In a Sybil attack, malicious nodes participate in a network not only with their own identity, but also present multiple other identities under which they act. For example, in voting or majority based systems, if left unchecked, this type of attack can allow an attacker to use a minority of nodes with many identities to overvote outvote the legitimate participants. Such attacks are very common in peer-to-peer networks, and they can threaten the overall security and integrity. Malicious or faulty agents that either by intent or accident act under multiple identities can end up subverting the system by assuming control of a substantial fraction of it [10].

Many previous works put their focus on the prevention of Sybil attacks by barring misbehaving nodes from entering the network [11,12]. Another common approach is to detect misbehaving nodes that act under multiple identities [13,14]. However, the main reason that such attacks are possible at all is the fact that there is no mechanism in place, which prevents the creation of (virtual) identities or nodes in the network. This is usually the case for ad hoc networks or other networks without access to a central authority that manages and restricts access to the network. Such a central authority would often require access to an internet connection that may not be available in the network, or it may be available only intermittently. We previously proposed the Unchained [15] as a different approach to this issue. Unchained economically disincentivizes the creation of new identities that could be used to deploy Sybil attacks. The approach is based on requiring a proof-of-work, but avoiding the necessity for the user creating the identity to perform this work by themselves. Instead, it is, in effect, offloaded to the mining network of a public blockchain and a direct payment transaction on that network is used to generate an identity creation proof that may be verified offline.

The original Unchained protocol and Rechained, as we propose here, both create identities from such transactions on a blockchain. Because these transactions are signed suing the public/private key pair of the sender, the identity also becomes tied to this key pair. The actual transaction that is used to create an identity has to follow certain requirements, such as a minimum amount of currency being transferred to one or multiple specific receiver addresses. We propose a way of determining certain amount boundaries, which ensure that attempting to circumvent the protocol would require the expenditure of more funds than are required by following it. Unchained focuses on offline verification of proofs, in effect “unchaining” its security mechanism and allowing its use in isolated networks with no internet connectivity.

This work builds on top of the initial Unchained publication [15] as well as a further extension, called UnchainedX [5]. In the following, we extend the Unchained protocol and its extension to incorporate the concept of self-sovereign identities (SSI) for network participants and add the missing functionality of revoking Rechained identities. Moreover, we formalize the protocol using Colored Petri Nets (CPNs) [16,17] to detect and eliminate possible design flaws, missing specification details, as well as thus far undetected security issues [18]. In Rechained, we also consider how scenarios with or with intermittent internet connectivity allow nodes to verify current blockchain parameters, which is also the reason for the updated name. Finally, we address the issue of eclipse attacks targeting the Rechained protocol.

The remainder of this paper is structured, as follows: Section 2 introduces supplementary literature and related work. Section 3 focuses on the operational details and outlines the security properties of the Rechained protocol. Next, Section 4 elaborates on different options to handle difficulty changes in the underlying cryptocurrency. In Section 5, we utilize Colored Petri Nets to create a formal model of our protocol. Afterwards, Section 6 presents an evaluation of Rechained based on case studies and the previously created CPN models. Finally, Section 7 concludes this work and provides an outlook on future work.

## 2. Supplementary Literature and Related Work

This section provides background information, supplementary literature, and it also introduces related work regarding previous approaches to solve the issue of Sybil attacks. Section 2.1 briefly summarizes the concept of self-sovereign identities and decentralized identifiers, while Section 2.2 provides general information on the concept of blockchain technology. Section 2.3 focuses on related work.

### 2.1. Self-Sovereign Identities and Decentralized Identifiers

Decentralized identifiers (DIDs) are a specific instantiation of the self-sovereign identity (SSI) concept. It has been proposed and it is also currently under development by the W3C [19]. DIDs provide a digital identity representation that is controlled by the owning entity while at the same time being “independent of any centralized registry, identity provider, or certificate authority” [19].

A DID (did:rechained:123456789abcdefghi) consists of three parts. First, the so-called URL scheme identifier (did), second the DID method identifier (e.g., rechained) and last the DID method-specific identifier (123456789abcdefghi). The scheme part simply explains that we are handling a DID. The DID method identifier defines “how a specific DID scheme can be implemented on a specific distributed ledger or network, including the precise methods by which DIDs are resolved and deactivated and DID documents are written and updated” [19]—in our case, the Rechained protocol. The last part of the example details the unique entity identifier.

A DID corresponds to an entity and resolves to a DID document, which is represented by JSON-LD documents and describes how to use the DID. The DID document consists of a reference that links it to the corresponding DID, public keys that can be used for verification purposes, authentication methods to authenticate a DID, or the owning entity and service endpoints [19]. Moreover, DID documents may contain an authentication property, a mechanism “by which a DID subject can cryptographically prove that they are associated with a DID” [19]. The authentication property provides a list of various verification methods, e.g., public keys. Proving control over a DID document is exerted by resolving the DID to a DID document according to its DID method specification. Proving control over the public key specified in a DID document is achieved via a signature-based challenge-response mechanism using the private key that corresponds to the public key.

### 2.2. Blockchain Technology

**Figure 1:**
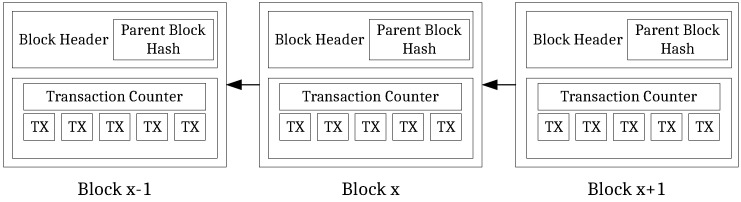
General blockchain structure—based on [20].

Figure 1 illustrates the general structure of a blockchain, as used by, e.g., the Bitcoin [20], or Ethereum platform [21]. As the name suggests, a blockchain consists of a sequentially ordered number of blocks that records transaction events (denoted as *TX*), e.g., transfer of a cryptocurrency from entity A to entity B. Each block contains the hash of the previous ancestor block, thereby chaining all blocks together. Changing a transaction in a block results in a hash mismatch of the succeeding block. As a result, tampering with one block requires the recalculation of all succeeding blocks.

Different methods for achieving a global consensus on which transactions are included in a blockchain exist. The most common approach is called proof-of-work (PoW), and it is in use by the most used blockchains Bitcoin and Ethereum [21]. proof-of-work in the way it is used in Bitcoin was first introduced as Hashcash [22]. In this approach, the so called mining process works by having many different parties attempt to solve a computational problem of variable difficulty. Specifically, a search problem is used, where the hash of a nonce value concatenated with the next block’s block header is hashed and a hash with a value falling below the predefined difficulty target number is searched. This means that the first miner to find a hash with beginning with a certain number of zero bits is allowed to publish the next block in the blockchain.

The important properties of this process are that the actual difficulty of the search problem can be adjusted over a wide range, while the result always remains easy to verify in constant time (a single hash operation). Increasing the difficulty target or number of leading zeros exponentially increases the average amount of work that is necessary to solve the problem [20]. Usually, each blockchain has a set target block time. This is the average time that it should take for a new block to be found. Using this as a target, the difficulty is adjusted to adapt the mining process to the amount of available computing power. If blocks are found too fast, the difficulty is automatically increased to compensate. If finding blocks takes too long, it is decreased. In the case of Bitcoin, these adjustments take place every 2016 blocks, which, with a target block time of 10 min., corresponds to around two weeks.

To incentivize the participation in the mining process, miners who find and publish a valid block may include a special transaction awarding themselves the so-called block reward, which also serves to initially distribute the currency. For Bitcoin, as of June 2020, this block reward amounts to 6.25 ₿ plus any transaction fees paid by users of the currency to have their transactions included in the blocks. Once a new block is published, all of the participants in the blockchain’s network will verify the validity of the block’s hash, its correspondance to the difficulty target as well the validity of the included transactions. Each block contains the hash of the previous block, chaining them together into a blockchain and ensuring that past blocks cannot be tampered with. As the longest chain, meaning the chain with the highest accumulated proof-of-work, is considered to be the valid chain, any attacker that would attempt to tamper with a past block would have to redo proofs-of-work for all subsequent blocks while being faster than the rest of the network, so as not to fall behind.

### 2.3. Related Work

Sybil attack prevention and Sybil attack detection in different network environments is a topic of on-going research with various approaches. Thus, we only highlight a selection of related work. SybilGuard [23] is a well-known protocol that limits the harmful influence of Sybil attacks in P2P networks. The SybilLimit protocol is an advanced version of SybilGuard and it aims to defend online social networks from Sybil nodes [24]. Both rely on human-established trust relationships; hence, they cannot be applied to mobile ad hoc networks or the IoT. Moreover, the correctness of SybilGuard depends on the fast-mixing property of the underlying social network graph, which constrains its applicability in IoT scenarios.

Other works focus on specific types of ad hoc networks. While [14] focuses on signal strength-based Sybil attack detection in MANETs, Ref. [25] targets the detection of Sybil nodes in VANETs. The latter deploys monitoring nodes that collect information, like distance, angle, or signal-strength, with neighboring nodes and use fuzzy logic on the collected data to detect suspicious nodes. While the presented solutions are applicable in some MANET or IoT networks, they have quite specific requirements, e.g., monitoring nodes, which limits their applicability and adds complexity to the network in terms of node configuration and computational tasks that are performed on the nodes. Refs. [26,27] try to prevent and detect Sybil attacks in sensor networks. The approaches are either limited to a particular application scenario (wildfires) or add the cost of additional complexity by deploying monitoring nodes. In contrast to the approaches that are presented above, Rechained focuses on preventing Sybil nodes from joining a network unless they sacrifice a pre-defined amount of resources instead of detecting them once they are already part of the network. Rechained does not require any monitoring nodes that collect data and perform extra computational tasks. Ref. [28] rely on a PoW-dependent Sybil node prevention strategy that requires nodes to calculate proofs-of-work to join a network, a solution that is rarely applicable in energy- and computationally-constrained IoT scenarios. The authors of [29] briefly outline some limitations of PoW-based Sybil node attack prevention and question the applicability of PoW for node identity generation. They argue that, by constraining the PoW difficulty so that users are willing to wait for it during identity creation, it will be limited to low costs that are not effective in disincentivizing Sybil attacks. Rechained, however, offloads the actual PoW process onto an existing Blockchain, such as the Bitcoin blockchain, which makes this argument inapplicable. Instead, the PoW process is replaced by a direct payment transaction on the blockchain.

Distributed ledgers and blockchains matured and spread in popularity—most noticeably by providing the foundation of the cryptocurrency Bitcoin [20]. Being inspired by the Bitcoin system, several further DLT platforms emerged, e.g., Ethereum (https://ethereum.org/, accessed: 5 May 2021), Hyperledger (https://www.hyperledger.org/, accessed: 5 May 2021), or Tezos (https://tezos.com/, accessed: 5 May 2021). Moreover, a variety of applications involving blockchain technology has been proposed, e.g., for IoT applications and platforms [30,31], in the automotive sector [32], in the agriculture industry [33], or for asset tokenization [34].

## 3. Protocol Specification

Our primary consideration is the creation of identities that can be verified without, or with only, irregularly available Internet connections. MANETs may be deployed in situations or environments without infrastructure, which facilitates Internet access for nodes. In IoT or vehicular applications, nodes may move through areas without connectivity or even remain there for a longer duration (e.g., underground parking without WiFi). In scenarios without, or intermittent Internet connectivity, identities are generated before the deployment of nodes and then preloaded onto them.

Specific applications further require resistance against eclipse attacks. In an eclipse attack, an attacker creates Sybil nodes with identifiers being selected to form a neighborhood around a target node according to some distance metric. In order to prevent this, the identifier of an identity should be selected randomly in such a way that the owner of the identity has no direct influence on the selection process. It becomes infeasible to perform such an attack if generating new identities is sufficiently expensive and there exists a sufficient number of nodes. We design our scheme with this in mind.

In the following, we present the design of our protocol, i.e., how to create and verify identities, as well as how our scheme ties into the concept of DIDs.

### 3.1. Creating Identities

**Figure 2:**
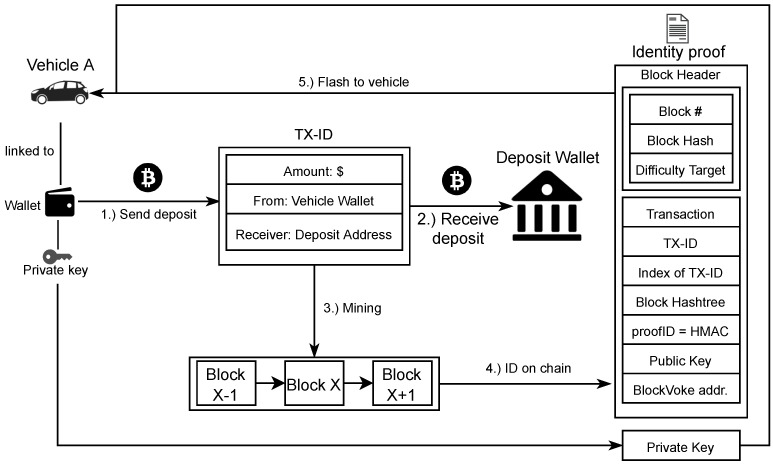
Creating a new identity proof—based on [5,15].

An overview of the binding process for an existing DID-based identity, e.g., did: rechained:123456789abcdefghi, is given in Figure 2. It assumes a pre-defined identity outside the Rechained context and a corresponding key pair that represents a wallet address. Alternatively, a non-DID based identity is instantiated without the DID linking by creating an identity using a public and private key pair that will be associated with the created identity and sends a transaction with a predefined amount to one or multiple predefined addresses. Once this transaction is mined, then the transaction and certain meta data will be used to construct an identity proof, which will allow network participants to confirm that the identity of the given public key was created legitimately. The created identity proof can finally deployed on a device to let it join the Rechained secured network.

We assume that a Bitcoin (or other cryptocurrency) address with public key pub and private key priv has been prepared for the process and has been funded with the necessary amount of tokens, e.g., Bitcoin. To explain the process, we first define a number of parameters.

receiver shall be the deposit address for our scheme. The deposit is sent to this address during the identity creation process. Possible attackers must not be able to recover the deposit from this address. In Section 4, we will detail various further properties of this address.

amount shall be the price of creating an identity. This is the minimum amount of cryptocurrency sent to receiver to create a new identity. More information regarding the choice of this parameter can be found in Section 4.

We follow the basic approach of BlockVoke [35] to make it possible to revoke this identity at a later point in time, which will be explained in more detail in Section 3.3. For this purpose, the user generates another separate Bitcoin (or other) address and also adds it to the identity proof.

To create a new identity, an amount of at least amount is sent to receiver from the address corresponding to pub (steps 1 and 2). Additionally, an OP_RETURN output is included in the transaction. OP_RETURN outputs are a way to include an arbitrary 40 byte payload in a transaction. In this case, it contains the address to be used for revocation purposes. The transaction is mined into block *x* of the blockchain (step 3). Subsequently, the block is used to create an identity proof (step 4) containing the block header (block number, block hash, difficulty target of the block, etc.), the deposit transaction, the Merkle tree proof hashes necessary to prove that the transaction is part of the block, the transaction index txIndex of the deposit transaction in the block, the public key pub, and a unique proof ID proofID of at least 128 bit. The proof ID is determined, as given in Equations (1) and (2).

(1) $${\mathrm{key}}_{\mathrm{HMAC}}:=x_{\mathrm{BlockHash}}$$

(2) $$\begin{array}{c} \mathrm{proofID}:=\mathrm{HMAC}({\mathrm{key}}_{\mathrm{HMAC}},\mathrm{txIndex}) \end{array}$$

Because the user creating the identity has no influence on the block hash or index of the transaction within the block, this way of calculating the proof ID makes it resistant to eclipse attacks. Even assuming that a user collaborates with a miner, influencing the resulting proof ID is costly, since it requires the miner to discard valid solutions for the PoW search puzzle in order to find a block hash and transaction index combination that satisfies any given requirements by the user.

The identity proof and private key priv are flashed or otherwise transferred to the device that uses the generated identity (step 5). The device can then be deployed or otherwise join the network.

### 3.2. Verifying Identities

**Figure 3:**
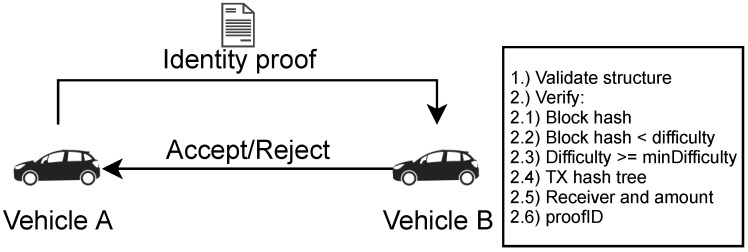
Validation process—based on [5,15].

Before nodes start communicating, they verify their peers’ identities to prevent Sybil nodes from entering the network for free. Figure 3 provides an overview of this process. To start communicating, two nodes exchange identity proofs as part of a two-way handshake. Alternatively, a node may have received an identity proof for another node through some other method, such as learning about it from neighboring peers.

First, the structure of the identity proof is verified to ensure that it contains the necessary block header, transaction data and index, Merkle tree proof, the public key, and the proof ID. Moreover, it is checked whether the public key matches the private key used during the two-way handshake.

For the next verification step, two configurable parameters minHeight and minDifficulty are used, which will be further explained in Section 4. If the block number from the block header is higher than minHeight, the block hash corresponds to a difficulty of at least minDifficulty, as well as the block’s own difficulty target, and matches the contents of the block header, this part of the verification process succeeds.

Next, the Merkle tree proof is used to verify that the given transaction is indeed part of the given block. It is also checked that the transaction sends an amount of at least amount to receiver and that its signature is correct and can be verified with the public key pub.

Finally, the proof ID is verified according to Equations (1) and (2).

If all of the verification steps succeed, the identity proof is accepted, and communications may take place. Otherwise, it is discarded, and no further communication takes place between the nodes.

### 3.3. Revoking Identities

It may become necessary to revoke identities, depending on the intended lifetime of the network. The most basic mechanism is to give each identity a limited lifetime. However, the drawback is that identities need to be recreated periodically, incurring unnecessary costs for users who have no reason to revoke their identities.

Therefore, we propose the following mechanism to revoke identities, basically following the approach of [35]. Each identity proof contains an additional Bitcoin address, as specified in Section 3.1. To revoke this identity proof, enough funds need to be sent to the revocation address to cover the transaction fees for an outgoing transaction. Thereafter, a Bitcoin transaction will be made from the revocation address containing only an OP_RETURN output containing the six bytes “RECHND” and the 32 byte transaction ID of the transaction originally used to create the identity proof. This transaction will then act as a proof of revocation that any node in the network can verify, if it knows the corresponding identity proof.

When creating their identity proof, users may scan the blockchain for revocations by scanning for transactions containing only an OP_RETURN output containing the six bytes “RECHND” and then verifying that the transaction that is specified by the transaction ID following these bytes could actually generate a valid identity proof following the usual verification rules. If this is the case, the proof ID may be added to a list of revoked identities and flashed on the node, together with the identity proof. A user may further include the most recent revocation transactions on the node and disseminate them through the network to help in preserving its integrity.

### 3.4. Distributed Identities

The previous Section 2.1 described the concept of decentralized identifiers (DIDs) as a specific instantiation of self-sovereign identities. Rechained may be integrated with DID-based SSIs in two ways. First, a Rechained identity proof is linked as part of the Authentication field of the DID document corresponding to the machine identity. The Authentication field contains verification methods authorized by the DID subject for authentication purposes. This way, the Rechained identity proof is used for authentication purposes.

Alternatively, we propose a standalone instantiation of Rechained by implementing a specific Rechained DID scheme, e.g., did:rechained:proofID. The generated proofID is used as the DID method-specific identifier and “did:rechained” indicates the implementation of a DID scheme implementation specifically for Rechained.

## 4. Parameter Choices and Updates

Rechained depends on a number of parameters that can be set up in different ways to make it suitable for different types and sizes of networks. Depending on the estimated value of an attack, the cost of identities can be adjusted to either make it easier for users to join the network or to make it costlier for attackers gain enough identities to perform attacks. Table 1 provides an overview of network parameters.

### 4.1. Network Parameters

In the following, we explain the configurable network parameters of Rechained.

#### 4.1.1. Starting Block Height

The parameter minHeight is used to set a certain block height as the minimum height acceptable to ensure that no identity proofs created prior to the creation of the network can be used. This also means that no significantly lower difficulty values need to be considered as difficulty tends to go up over time, as shown in Section 6.

#### 4.1.2. Deposit Address

The parameter receiver is the address that funds have to be sent to in order to create a new identity. The main property of this address is that an attacker trying to create a large number of identities is unable to recover the funds from it in any way. A receiver address has to be used rather than using the funds as mining fees, since an attacker may be able to collude with a miner or successfully mine a block themselves, allowing them to recover mining fees. In the following, we present three options for a receiver address.

The most secure way is proof-of-burn [36]. To burn funds, they are sent to an address with no knowable existing private key. However, because this method destroys the funds and the supply of Bitcoin is limited, it is considered to not be an elegant solution.

If the network secured with Rechained is developed or maintained by a certain entity, this entity may provide a receiver address under their control, i.e., the network operators sell identities. The funds thus gained are used for further development and maintenance or simply be considered as profit. Unless there is a conceivable reason for the network operators to attack their own network, this should also prevent possible attackers from recovering funds after the creation of identities.

Finally, receiver could be the donation address of a charity. In fact, it would be possible to slightly extend the scheme in such a way, where receiver contains multiple donation addresses and funds that must either be distributed equally among them or sent to one of them according to the user’s choice. Unless the chosen charities all have an interest in attacking the network or are compromised in some way, an attacker is highly unlikely to be able to (fully) recover the funds.

#### 4.1.3. Deposit Amount

The parameter amount sets the minimum amount that has to be send to the receiver address. The value is chosen in such a way that it is high enough to disincentivize the creation of spurious or malicious identities, while still being affordable for regular users who wish to participate in a given network. The choice of amount may also be influenced by the expected size of the network, as smaller networks may be more vulnerable against attacks and may, therefore, require a higher amount setting to defend against them. At the same time, the expected value that can be gained from attacking a network with Sybil identities should also be considered when setting the amount parameter.

Alternatively, Rechained can be implemented in such a way that a smaller amount is sent to receiver, while a second bigger amountLocked is sent back to the user creating an identity. However, the output amountLocked is time locked for the expected life time of the identity using a CheckLockTimeVerify [37] output. As a result, users recover most of their funds once they stop participating in the network, while still requiring attackers to acquire a significant amount of funds up front.

Further considerations on the choice of amount are given in Section 4.2.

#### 4.1.4. Minimum Difficulty

The parameter minDifficulty represents a set of different parameters, depending on the current block height. It is initially set at the time the network is created to the current difficulty, or slightly lower to compensate for fluctuating difficulty values. Unless overwritten by some sort of update mechanism, as discussed in Section 4.3, this remains the minimum accepted difficulty for identity proofs. If an update mechanism is implemented, then each period of time between difficulty adjustments (2016 blocks in Bitcoin, or roughly two weeks) will receive its own minDifficulty value.

### 4.2. Price Considerations

We attempt to disincentivize the creation of Sybil identities by introducing cost to the creation of identities. Any attacker would attempt to minimize their cost of attack. In networks without Internet connectivity and rare network parameter updates, an attacker may attempt to do so by making use of the actual Bitcoin network’s target difficulty and the presumably lower target difficulty still used by Rechained. Such an attack is performed by cooperating with a miner to create a block with a target difficulty minDifficulty, filled completely with identity creating transactions. As this block targets a lower difficulty than the target difficulty of the Bitcoin network, it is cheaper to mine, but it cannot be published to earn block rewards. This introduces a significant opportunity cost for any miner cooperating with an attacker, unless the difference in target difficulties is very large.

An attacker has to compensate the miner for at least the opportunity cost value and, most likely, also has to pay an additional fee. The opportunity cost of creating a block and not publishing it is easy to quantify, as it is equal to the block reward (6.25 ₿) and any additional fees paid by transactions. Given the block size limit of 1 MB and a minimum transaction size of 224 B, this leads us to an upper limit for identity prices as calculated in Equations (3) and (4). Above these identity prices, the opportunity cost of creating a block optimized for identity creation may fall below the amount that is spent to create these identities properly.

(3) $$\begin{array}{cc} {\mathrm{amount}}_{\max} & =\mathrm{block}\;\mathrm{reward}\times \frac{\min\;\mathrm{TX}\;\mathrm{size}}{\max\;\mathrm{block}\;\mathrm{size}} \end{array}$$

(4) $$\begin{array}{cc} & =6.25\;\mathrm{BTC}\times \frac{224\;B}{1\;\mathrm{MB}}=0.0014\;\mathrm{BTC} \end{array}$$

As of 22 April 2021, the price of Bitcoin is at approximately 52,009 USD [38,39], leading to a maximum identity price of approximately 72.81 USD.

In networks with at least intermittent Internet connectivity, an attack, as described above, is unlikely to occur as nodes can easily check if blocks are actually part of the blockchain once they get online. To do so, the nodes neither have to download the full blockchain nor have light-client capabilities. Instead, they act like super-light clients that just query a trustworthy source of their choice for the relevant block hashes. The calculations regarding the amount may still be a valuable guideline for pricing identities.

#### Blockchain-Based 51% Attacks

At this point, the possible impact of blockchain-based 51% attacks, where one party controls more PoW hashing power than the rest of the network, should also be considered. Generally, this kind of attack can be considered to be a catastrophic event for the underlying network and undermine its security and public trust. In the case of Rechained, performing a 51% attack would be a highly costly path of attack, as compared to the aforementioned methods. However, if such an attack is already taking place, an attacker might be able to use such an ongoing attack to create identities more cheaply. It is expected that the price of a cryptocurrency undergoing a 51% attack would decline sharply, in turn reducing the costs of creating identities. It might also become possible for an attacker to bribe the party performing the 51% attack to let the attacker double-spend the funds used for identity creation.

### 4.3. Updating Parameters

For scenarios where network participants have Internet connectivity at least sometimes, nodes may check the difficulty at certain block heights and set their corresponding minDifficulty accordingly. The amount parameter is updated either through signed updates published by the network operator or one of the mechanisms discussed in the following, except that there is no need to distribute these updates in a P2P-manner.

In scenarios without Internet connectivity, the following mechanisms are proposed to keep the network parameters up-to-date and minimize the impact of difficulty and price changes. They may also be used in scenarios with intermittent Internet connectivity to bridge the offline periods.

#### 4.3.1. Maximum Seen Difficulty

This is a fully decentralized approach that is also easy to implement. Once a node first receives an identity proof created within a given, so far unknown, difficulty adjustment period of two weeks, it sets the target difficulty seen in that identity proof’s block as the minDifficulty for this period if it is above the networks initial minDifficulty value. If an identity proof is received for a period that has been initialized in this way, and the new proof’s block contains a target difficulty that is higher, any previous identity proofs for this period with a lower target difficulty are invalidated and the minDifficulty for the period is updated to the new value.

This method allows for the eventual detection of identities that are created using forged lower difficulty blocks once a connection to an honest node whose identity was issued in the same two weeks period is made. It does not require any additional infrastructure to operate.

However, the approach is vulnerable to a denial-of-service attack. If an attacker creates a block for a two weeks period that has a higher difficulty than the target difficulty of the actual Bitcoin network, all of the honestly created proofs will be discarded. As creating such a block is even more difficult than creating a regular Bitcoin block, and it also cannot be transmitted to the Bitcoin network due to a mismatch in the target difficulty block header field, this attack incurs significant costs for the attacker.

The method can be used as a fallback method for the following approaches.

#### 4.3.2. Bundled Updates

If the network is run by a single operator, this network operator may publish signed network parameter messages for every difficulty adjustment period. When creating their identity proofs, users also download the corresponding update message. When joining the network, the user’s node distributes the update message together with the identity proof. Nodes receiving the update message verify the signature and set their minDifficulty and amount for the given period accordingly. This can be combined with the previous approach to allow nodes to join without having to retrieve an update bundle. A minDifficulty set through an update message would never be overridden by a higher difficulty, thus preventing denial-of-service attacks. At the same time, combining both methods still allows users to keep joining the network through the maximum seen difficulty approach if the network operator ceases publishing update messages.

#### 4.3.3. Majority Vote

A more decentralized alternative to the previous approach is for nodes to accept signed difficulty update messages from multiple providers. Any number of these messages may be provided together with an identity proof. The difficulty values are stored in a list for each difficulty adjustment period. If different values are provided by different providers, the majority vote is treated as the correct difficulty value. If there is no majority, then the highest value is treated as the correct difficulty value.

The approach removes the single-point-of-failure that is present in the previous proposals above. If an attacker wants to influence the target difficulty for a given period, then the attacker has to compromise a majority of update providers to get their difficulty target chosen. Like bundled updates, this approach can be used with the maximum seen difficult approach as a fallback option for nodes that are unable to provide bundled update messages or in the case these messages cease being available at some point.

## 5. Protocol Formalization

Designing and specifying a new security protocol, such as Rechained, is a difficult task. Designing the protocol in such a way that prevents design flaws, security issues, as well as incomplete specifications that pose risks to the protocols stakeholders and the user is yet another challenge [40,41,42,43]. Even in a best-case scenario, issues of a security protocol can pose dangers to the individual users who rely on it, while, in other cases, design flaws and errors bring about serious real world consequences: The broken encryption of a wireless network [44] is an example for the first case, whereas a broken security protocol that grants an attacker access to sensible parts of nuclear power plants [45] illustrates a more serious threat.

Formal methods, such as Petri nets [46], π-calculus [47], and communicating sequential processes [48], address the posed challenge and are utilized for the design, development, and analysis of new as well as existing protocols, thereby eliminating, or minimizing, the security issues of the targeted protocols [49,50].

### 5.1. Colored Petri Nets

In the following sections, we formalize the Rechained protocol using Colored Petri Nets (CPNs) [16,17] in order to detect and eliminate eventual design flaws, missing specification details, as well as thus far undetected security issues [18]. CPN is a graphically oriented language that is used to design, specify, simulate, as well as verify systems. Moreover, it allows for describing the states of a modeled system and the events that cause the system to change states.

CPN models are represented using a directed bipartite graph that consists of places, transitions, arcs, and tokens. Places are denoted as circles and transitions as rectangles. Arcs connect places with transitions, or transitions with places, and have inscriptions given as CPN-ML expressions [16,17,51,52,53]. CPN-ML is an expression programming language for inscriptions that are used to further specify data types and operations of the modeled system. CPN tokens and their colors represent the different data types of the modeled system. The resulting CPN model “of a system describes the states of the system and events (transitions) that can cause the system to change state. By making simulations of the CPN model, it is possible to investigate different scenarios and explore behaviors of the system” [16].

Besides its general suitability for system formalization, CPNs are especially well-suited for application in the context of blockchain-based systems. CPN models are discrete state machines and change states via transitions. Analogous to this, blockchains are also discrete state machines as well, where the most recent block represents the current state of the system. With each new block, the system’s state transitions to a successor state. While, in CPNs, data structures are represented in the form of colored tokens, many blockchain platforms also use a data structure concept of tokens for the same reason. Moreover, blockchain transactions can be easily mapped to CPN token data structures. Furthermore, CPNs use CPN-ML expressions to specify and implement data types and operations of the modeled system, which correspond to the functionalities of smart contracts in the context of blockchain technology. Finally, the hierarchical structure of CPN models can be used to formalize blockchain-based dApps (decentralized applications) components of interleaved smart contracts. Thus, CPNs are well-suited as a formalism of choice for blockchain systems.

In the following, CPN-Tools (http://cpntools.org/, accessed: 5 May 2021) is used to design, evaluate, and verify the CPN models. The result is a formal specification of the protocol that is used to guide further implementation efforts and design decisions.

### 5.2. Modeling Strategy

An appropriate modeling strategy is required to map the existing descriptions of the Rechained protocol as described in Section 3 and Section 4 to the corresponding elements of a CPN model. We map the informal descriptions and requirements of Rechained to a formal model using CPN, resulting in a sound formal model. To do so, we first outline the modeling strategy used to create the CPN models before presenting the resulting CPN models in the subsequent sections.

Rechained organizes and defines the exchange of information between different entities that are modeled as agents. In software engineering, various agent-oriented approaches exist, such as: Tropos [54], Gaia [55], Prometheus [56], MASB [57,58], and MaSE [59]. In [60], Mahunnah et al. introduce a mapping heuristics from agent models to CPN models based on Sterling’s and Taveter’s [61] sociotechnical requirements-engineering methodology of Agent-Oriented Modeling (AOM).

In system development and software engineering, good requirements follow certain characteristics. Requirements address one issue only and they are completely specified without missing information, according to [62,63]. Moreover, they have to be consistent and must not contradict themselves, or pose contradictions in correlation with other requirements. Finally, a requirement must also be atomic and without conjunctions [64].

**Figure 4:**
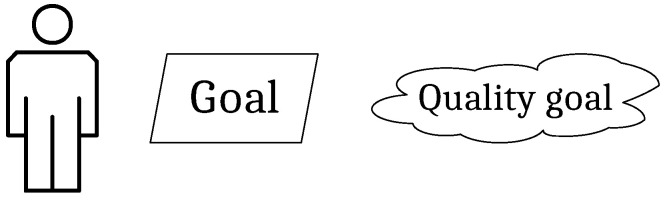
Selection of AOM notation elements.

The Agent-Oriented Modeling (AOM) methodology allows technical- and non-technical stakeholders to model complex systems by capturing and understanding their functional- and non-functional requirements. An AOM goal model relies on three main elements to capture the system requirements and goals, as illustrated in Figure 4. Involved stakeholders are represented as sticky men, being usually used solely for human entities, but this work also comprises IoT devices, agents, and infrastructure components. Parallelograms depict functional requirements and are referred to as goals. Non-functional requirements are depicted as clouds and refer to as quality goals of the modeled system. The AOM goal model follows a tree-like hierarchy with the root value proposition of the modeled system at the top. Subsequently, this main goal is decomposed into sub-goals, where each sub-goal represents an aspect for achieving its parent goal [65]. The goals are further decomposed into multi-layered sub-goals until the lowest atomic level is reached. Additionally, the roles and quality goals may be assigned to goals and they are inherited to lower-level goals.

### 5.3. AOM Model

**Figure 5:**
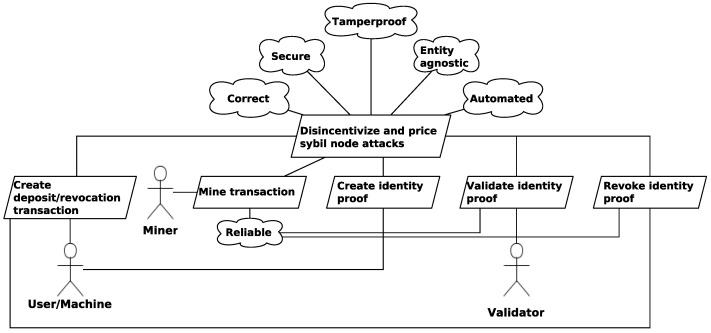
Rechained top-level AOM goal model—extension of [5].

Figure 5 presents the AOM goal model of the Rechained protocol.The main objective of Rechained is to disincentivize and price the cost of Sybil node attacks. The main goal is further decomposed into multi-layered sub-goals until the lowest atomic sub-goal is reached. In the context of Rechained, the main goal is further divided into the following sub-goals: *Create deposit transaction*, *Mine transaction*, *Create identity proof*, *Validate identity proof*, and *Revoke identity proof*. The five quality goals *secure*, *correct*, *tamperproof*, *entity agnostic*, and *automated* are attached to the overall main goal of the goal model, meaning they are relevant and inherited to all sub-goals. The quality goal *reliable* pertains to the three sub-goals of *Mine transaction*, *Validate identity proof*, and *Revoke identity proof*. In addition, we list three different roles: The *user*—either a human, or machine—the *mining* entity that performs the PoW calculations of the underlying blockchain and the validator who validates an identity proof once received.

Next, the AOM behavior model refines the AOM goal model for specific agents and activities. A behavior model in AOM has two parts: An agent behavior model is coupled with a behavior interface model [61]. The former describes the rule-based behavior of an agent, while the latter focuses on identifying activities with associated triggers, preconditions, and post-conditions [60].

Table 2 presents the behavior interface model of the goals that are depicted in the goal model of Figure 5. Each activity is listed with its corresponding trigger, optional pre-conditions, and its post-conditions. The execution of an activity is either triggered by an event, or by a pre-condition after the occurrence of an event [60].

The *Create Deposit Transaction*-activity is triggered after providing the required network input parameters as well as a machine identity (e.g., a network node) and a wallet. Afterwards, as part of the *Mining*-activity, the resulting deposit transaction is mined into a new block. During the *Create Identity Proof*-activity, the block, the machine identity and the provided wallet are used to create an identity proof for the node. Finally, the created identity proof is validated as part of the *Validate Identity Proof*-activity that takes an identity proof, the network parameter, the machine identity, and the initial wallet to determine whether the identity proof is valid.

### 5.4. Mapping AOM Models to CPN Models

**Figure 6:**
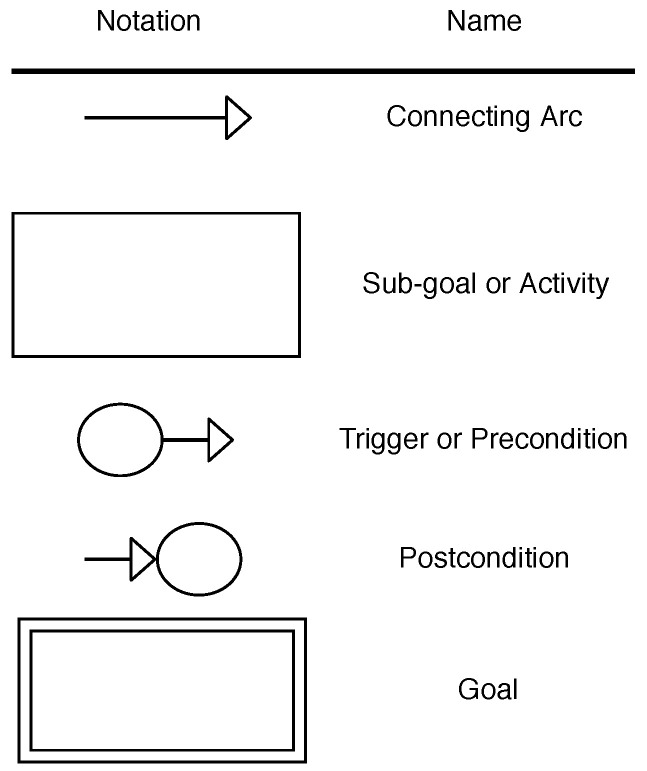
Notation mapping CPN to AOM—based on [60].

**Figure 7:**
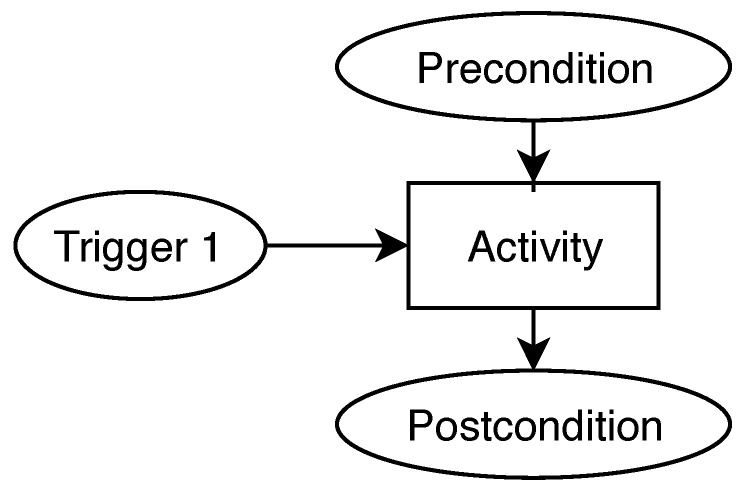
Mapping a behavior interface model to a CPN model—based on [66,67].

Mapping the created AOM goal and the AOM behavior interfaces to CPN is the final step necessary to derive a CPN model of Rechained. Figure 6 and Figure 7 illustrate the mapping heuristic of AOM goal models to CPN models as well as the mapping of behavior interface models to the CPN models.

In the CPN model, the Rechained protocol execution is modeled using places and transitions thata re connected by directed arcs. The goals of the AOM goal model are mapped to rectangular CPN transitions. Double-boarded transitions are used to indicate sub-goals and hierarchically structured CPN modules. The CPN modules and sub-modules of the overall CPN model map to the relation between goals and sub-goals of the AOM goal model, as illustrated in Figure 7. The triggers and pre-conditions of the AOM behavior interfaces are displayed as places with outgoing arcs, while post-conditions are represented by places with incoming arcs [60].

**Figure 8:**
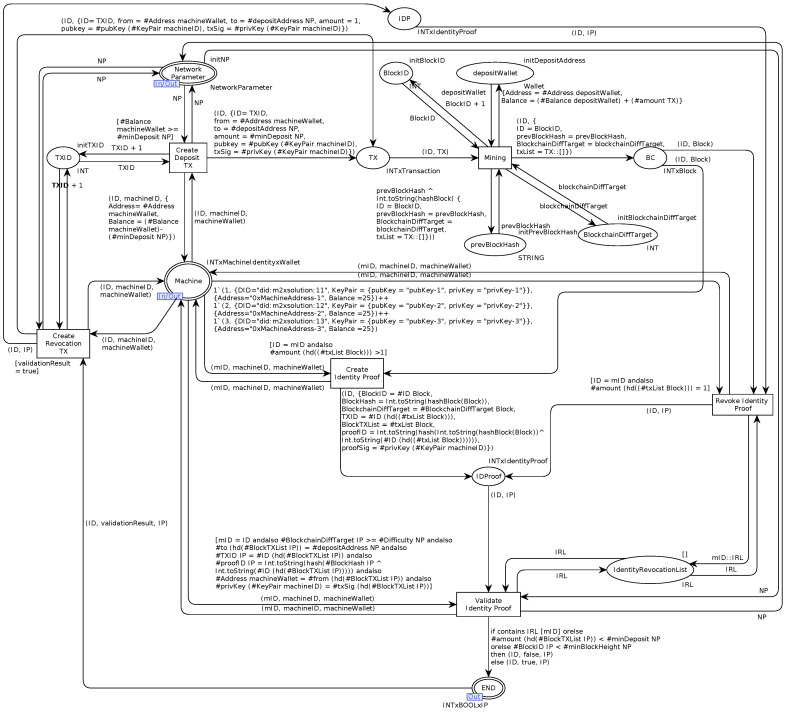
Rechained CPN model—extension of [5].

The complete and formalized Rechained CPN model as derived from the AOM goal model and the AOM behavior interfaces and implemented using CPN-Tools is shown in Figure 8.

The CPN model consists of six transitions that are derived from the five sub-goals of the top-level AOM goal model. The protocol flow starts on the left-hand side of Figure 8 with the *Create deposit transaction*-transition. An infrastructure provider, user, or machine that wishes to create a new identity triggers the transition by providing the required network parameters, as described in previous sections, as well as information relating to the entity’s identity (in the CPN model, a machine is assumed), i.e., a machine identity consisting of a DID and a public/private key in addition to a wallet that corresponds to the used key pair. In case the wallet balance is sufficient to make a deposit, a matching deposit transaction with the target deposit address is created via the *Create deposit transaction*-transition. After that, the transaction is mined into a new block of the underlying blockchain platform (*Mining*-transition). The resulting block contains a BlockID, the hash of the previous block, the blockchain’s difficulty target, and a list of included transactions. Once the block is mined, an identity proof is created (*Create identity proof*-transition) according to the specifications described in previous sections. Next, the resulting identity proof is checked for validity while passing the *Validate identity proof*-transition resulting in a Boolean representation of the validation process’s success or failure. Subsequently, a valid identity proof may be revoked by the owning entity. To do so, the *Create revocation transaction*-transition creates a revocation transaction similar to the deposit transaction, which is mined into the blockchain. Afterward, the *Revoke identity proof*-transition allows for revoking an identity proof, registeriing the revoked proofID with the *IdentitiyRevocationList*. Once revoked, the identity proof validation fails and the CPN model terminates.

The CPN token color sets, names, and abbreviations used in the model are introduced in the following sections, while the complete and executable CPN model is available in Data Availability Statement.

### 5.5. Protocol Semantics

Next, we introduce the CPN token color sets, names, and acronyms of the Rechained CPN model. CPN token colors represent the data structures of data objects that are used to illustrate the data flow throughout the CPN model. Exemplary acronyms, names, and the description of the token colors of the CPN model are presented in Table 3. The first column specifies the name, followed by a short description in the second column. The last column lists information concerning the data types. A complete list of all acronyms, names, and abbreviations, as well as the description of the token colors of the Rechained CPN model is available in Data Availability Statement.

## 6. Evaluation and Discussion

The following section focuses on evaluating the Rechained protocol. First, Section 6.1 and Section 6.2 focus on the evaluation of the security guarantees that are provided by Rechained. Those mainly depend on the target difficulty level as well as the token price of the underlying PoW blockchain. In the context of this work, we choose the Bitcoin and the Ethereum blockchain as the most popular and utilized PoW chains to deploy a fictional network of Rechained nodes. The combination of evaluating both blockchains covers important corner cases of changing difficulties and token prices, such as increasing and decreasing difficulty in combination with sudden price declines and raises. Our evaluation focuses on the time period from January 2017 to April 2021. Even though neither future price developments of Ether and Bitcoin nor the target difficulty can be reliably predicted, the analysis provides an intuition on the historical worst-case performance of Rechained.

Afterwards, Section 6.4 presents the results of the state-space analysis of the Rechained CPN model. Subsequently, Section 6.5 presents an Ethereum- and Bitcoin-based proof-of-concept implementation. Finally, Section 6.6 critically discusses the evaluation results.

### 6.1. Bitcoin Price and Difficulty Analysis

**Figure 9:**
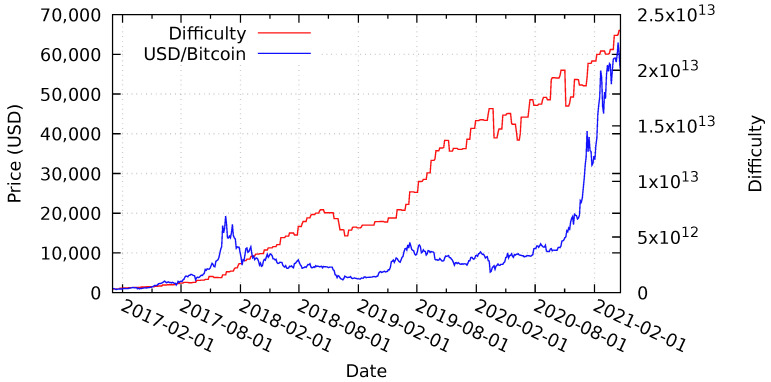
Average daily price of Bitcoin in USD and block difficulty level between January 2017 and April 2021—partially based on [5,15], Data Source [39].

Figure 9 presents the target difficulty level and token price of the Bitcoin blockchain between January 2017 and April 2021. The target difficulty level is steadily increasing with minor decreases in October 2018, October 2019, and March 2020—the last one being caused by the global COVID-19 pandemic. The price of the Bitcoin token follows a similar pattern, but with a high-price peak at the end of December 2017 and higher volatility. The lowest token price (ignoring the low price at the beginning of our evaluation period)—caused by the largest price-drop—occurs in January 2019. Very recently, throughout the beginning of 2021, the price has spiked upwards again significantly.

Section 4 discusses the lower bound security guarantees of Rechained, which depend on the minimum target block difficulty and the lowest token price equivalent per block that occurred during the existence of a particular network. Figure 9 shows that all of the nodes joining a hypothetical network later than January 2017 have higher security guarantees than the initial bootstrapping nodes due to an increased block difficulty and token price—even despite substantial declines of the Bitcoin price and the target difficulty levels between the end of 2017 and the end of 2018, as well as a subsequent decline in early 2020; the lowest price and difficulty levels still remain above the values at the time of the network initialization. Nonetheless, since the minimum price per identity is determined by the network operator, the token price and difficulty level only represent a theoretical measurement for security guarantees. Yet, a higher token price results in an increased block price, which again results in higher attack costs.

Deploying a network at the lowest price of our evaluation period results in subsequently higher security guarantees after price gains. However, deploying a network at the highest point in Bitcoin’s price history, at the beginning of November 2017—days before the decreasing Bitcoin price as illustrated in Figure 9—decreases the security guarantees, and makes it less expensive to introduce new identities into the system for a short period of time. Again, the actual pricing of identity proofs depends on the network operator determining the minimum price of an identity. For practical reasons, it is likely that most of the operators pick minimum values below the maximum determined in Section 4.2, allowing for difficulty declines without affecting the security guarantees of a given network. In case of substantial price and difficulty declines, e.g., between the end of 2017 and the end of 2018, a price and difficulty network parameter update is recommended.

Upwards price spikes, like in December 2017 and the beginning of 2021, can present a concern with regards to the usability of a network using Rechained. A sharp increase in price will also increase the costs to join the network. If a network is created during a price spike, the operator may elect to configure the required amount to be lower to compensate, but, if a network is created and configured before such an increase, it may cause issues. To address this, one of the proposed network parameter update mechanisms from Section 4.3 should be used. Overall, higher prices are beneficial to the security of Rechained, as they allow more freedom and a higher upper bound of the dollar price per identity when choosing a price per identity as per Equation (3). At the same time, it also remains possible to choose lower prices per identity.

Figure 9 only provides an overview of the volatility of the Bitcoin token price and the block difficulty level. Table 4 utilizes the same data set and analyzes the occurrence of substantial token price declines by calculating, for each possible deployment date of a hypothetical network, the highest drop in price and, thus, security level experienced by the network. Price drops are calculated based on the daily average Bitcoin prices at the end of each given day. A price lower than the previous day indicates a price decline while the opposite is true for increasing prices. The probability of dropping, at any point in time during the evaluation period, below 10% of the initial security level is 0%, while there is only a 1% chance of dropping below 20% of the price at any point in time.

Smaller price-drops occur frequently. Almost 76% of all starting days experience price drops of at least 10% at some point in our analysis. However, substantial declines are rare. Most of the networks can tolerate some price volatility without relevant declines with regards to the provided security level. Nonetheless, large price drops or rising token prices are an issue. While price drops result in lower security guarantees, rising prices may be an issue too since they can make identities too expensive for regular users. Thus, an update mechanism for the network parameter amount is recommended in the context of networks that operate for a long time. We have proposed different mechanisms to perform such updates in Section 4.3. Even though price volatility is a major issue for most cryptocurrencies right now, the volatility level of cryptocurrencies and fiat currency is expected to converge if the mass-adoption of cryptocurrencies occurs. As a result, Rechained’s security guarantees could be expected to vary less, due to the lower price volatility.

### 6.2. Ethereum Price and Difficulty Analysis

**Figure 10:**
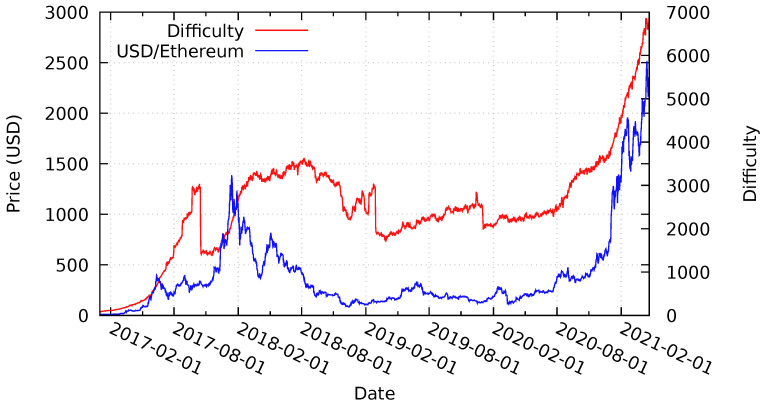
Average daily price of Ether in USD and block difficulty level between January 2017 and April 2021—partially based on [5,15], Data Source [69].

Rechained itself is a blockchain-agnostic protocol. It only requires the underlying blockchain platform to utilize a PoW-like consensus algorithm. Therefore, we also analyze the provided security guarantees for hypothetical Rechained networks when deployed on the Ethereum platform during the same time period as above. Figure 10 illustrates the Ether token price and the Ethereum block difficulty between January 2017 and April 2021. Similarly to the Bitcoin token price, the Ethereum token price also increased massively between January 2017 and the end of December 2017, followed by a significant drop until April 2018. Subsequently, a price recovery in May 2018 is followed by a further decline until December 2018, before slowly starting to increase again until February 2020, followed by a decline that is caused by the COVID-19 pandemic at the end of the evaluation period and another steep incline at the beginning of 2021. The block difficulty increases steadily until 16 October 2017, before a sudden drop due to a difficulty adjusting hard-fork of the Ethereum network [68]. Another drop is shown at the end of 2019. Despite substantial reoccurring declines of the block difficulty level, even the reduced values were higher than the initial level in January 2017. During the steep incline in price in 2021, the difficulty also increases accordingly. It appears that, in the case of Ethereum, the difficulty is more closer linked to price than in the case of Bitcoin, where it increased steadily, even through a period of lower prices.

The overall behaviour is similar to Bitcoin. Hence, the security guarantee evaluation results are similar to the evaluation of the hypothetical Bitcoin-based Rechained network assumed in Section 6.1. Network nodes deployed in January 2017 with the bootstrapping difficulty level and token price are cheaper and easier to create in terms of identity price and block difficulty. All of the nodes deployed at later points in time provide higher security guarantees. Identity proofs created briefly before and/or after the Ethereum difficulty adjustment, as well as the difficulty drop in early 2019 and late 2019, are less difficult to create than identities created later on. The same applies for the price of identities both before and after the price declines of Ether in 2018, as illustrated in Figure 10. Because Ethereum seems to have sharp drops in difficulty more often than Bitcoin, it seems prudent to choose the initial minDifficulty significantly lower than the current difficulty, to account for such drops in the future, unless timely network parameter updates can be guaranteed. With respect to the recent increase in price with the beginning of 2021, the same considerations as with Bitcoin also apply.

Analogously to Table 4, Table 5 analyzes the occurrence of substantial token price declines for the Ethereum network by calculating, for each potential deployment date of a hypothetical network, the highest drop in price and, thus, the security level experienced by the network. The probability of dropping, at any point in time during the evaluation period, below 10% of the initial security level is 3.3%. Drops below 20% has would have occurred for 19.6% of possible starting dates. Almost half of the affected dates experience price drops of 50%. These values are much higher than the values for the Bitcoin network, as presented in Table 4. Thus, the Bitcoin-based Rechained solution provides more certainty and more steady security guarantees than the Ethereum-based implementation—at least based on the analysis of past events, which is, of course, not indicative of future prices and volatility.

### 6.3. Transaction Fee Analysis

Storing data on a PoW-base chain, such as the Bitcoin blockchain, incurs costs that correlate with the transaction size. A revocation transaction in the Bitcoin network with a payload of 40 bytes has a size of fewer than 283 bytes, as suggested in [35]. Because Rechained does not depend on fast transaction processing, it is unnecessary to aim for a high mining priority by paying high fees. In March 2020, the cost of a transaction averaged around $0.001751 per byte. As a result, a revocation transaction of 283 bytes on the Bitcoin platform cost $0.496 [35]. However, the most recent Bitcoin hype increased the transaction fees heavily. In March 2021, the cost for the same revocation transaction increased to $6.281, with an average price of $0.022194 per byte [70,71].

Despite the drastic increase in transaction costs, we do not expect any long-term disadvantages for Rechained for several reasons. First, Rechained does not explicitly rely on the Bitcoin blockchain. Instead, all of the PoW-based blockchain platforms can be used as an alternative. Second, we expect the transaction fees to decline in the long run, either due to an end of the hype or as part of the widespread adoption of Bitcoin as a payment processing network. Finally, high transaction fees are addressed in various proposals that aim to reduce the fees, e.g, [72,73].

### 6.4. CPN State-Space Analysis

Next, we evaluate the Rechained CPN model by performing a state-space analysis, which we use to derive model properties and explain their implications. A state-space calculates a directed graph of all reachable states and state changes of a given CPN model. The nodes of the graph correspond to the set of reachable markings, and the arcs correspond to occurring binding elements [17]. The properties of the CPN model—and the system presented by the model—are deduced from the resulting graph. The state-space analysis used in this work is generated using the built-in functionalities of CPN-Tools. Subsequently, we calculated the SCC (strongly connected component) of our CPN model based on the state-space analysis’s previously generated directed graph. The nodes of the SCC are “obtained by making a disjoint division of the nodes in the state space such that two state-space nodes are in the same SCC if and only if they are mutually reachable, i.e., there exists a path in the state space from the first node to the second node and vice versa” [17]. Properties that are derived from the SCC may imply one or more cycles in case the SCC contains fewer nodes than the state-space graph of the CPN model.

The state-space analysis results and selected properties derived from the analysis of the Rechained CPN model are presented in Table 6, while the complete state-space analysis is available in Data Availability Statement.

None of the tested modules contain any loops, as shown in Table 6. Thus, no infinite occurrences of execution paths in the state-space graph exist, which guarantees the termination of the model. The state-space analysis also shows the absence of any home markings. A home marking is a marking that can be reached from any other reachable marking, meaning that it is impossible to have an occurrence of a sequence that cannot be extended to reach the home marking. The occurring dead markings are caused by customized input values that prevent a state-space explosion. “A dead marking is a marking in which no binding elements are enabled” [17]. The existence of at least one dead marking guarantees a termination of executable actions at a certain point, thereby preventing infinite runtime. The existence of a dead marking implies that the CPN model does not have a live transition. “A transition is live if from any reachable marking we can always find an occurrence sequence containing the transition” [17]. Finally, the state-space analysis did not yield any occurrences of dead transitions. A transition is considered to be dead if there is no reachable marking that enables the transition. Therefore, in the context of the Rechained CPN model, all transitions of the model can be potentially enabled at a certain point during the protocol execution [17].

### 6.5. Proof-of-Concept Implementation

Previous sections evaluated Rechained with regards to the provided security guarantees based on the underlying blockchain platform and cryptocurrency. Moreover, a state-space analysis using a formal CPN model was conducted. However, the real-world applicability and feasibility within the context of IoT devices have not been covered yet. Thus, a proof-of-concept implementation of the original Unchained protocol based on the Bitcoin and Ethereum blockchain was created—the repositories are available online (https://github.com/bleidingGOE/unchained-cli-btc or https://codeocean.com/capsule/7153326/tree/v1; https://github.com/bleidingGOE/unchained-cli-eth; or, https://codeocean.com/capsule/1342035/tree/v1, accessed: 5 May 2021). The implementations use public/private key pairs instead of DIDs thereby reduce development overhead and do not yet contain revocation support. However, this does neither affect provided security guarantees, nor the performance of the proof-of-concept. The implementations are evaluated using common IoT hardware, i.e., the Raspberry Pi 3 platform.

The size of an identity proof varies depending on the blockchain platform as well as the size of the block containing the proof transaction. Bitcoin-based proofs require between 10–50 KB of storage and they can be verified in about two seconds using a Python based implementation. Both runtime and proof size could most likely be optimized further for actual use in production. The Ethereum proofs consume around 50–150 KB of storage, but take around 60 s to be verified. The slow verification processing is caused by a deliberate design choice of the Ethereum hash function Ethash [74], which was designed to achieve ASICS resistance. Therefore, deploying Rechained on a Bitcoin-like PoW blockchain is more practical even though other PoW blockchain platforms that do not rely on Ethash or similar algorithms with the same property are also suitable.

Because the identities are deployed to networks by the network operators or device manufactures, we assume that the proofs itself are not generated on the actual devices—hence, we do not conduct any performance benchmarks for creating proofs on the Raspberry Pi 3.

### 6.6. Discussion

While the integration of DIDs in the context of Rechained allows for an effective mechanism to disincentivize and accurately price Sybil node attacks, the presented solution still misses a comprehensive integration into industry-standard implementations, e.g., for vehicle-specific use cases, the recently presented vehicle identity standard [75]. Integrating the vehicle identity standard, which uses a DID-structure, with Rechained identity proofs is desirable.

Evaluation limitations of the Rechained CPN model result from the customized input statements of the model as well as the modeling process itself, which requires several simplifications, e.g., neither the Bitcoin nor the Ethereum consensus algorithm and mining process were implemented in the CPN model. Furthermore, we simplified the data structures of the Rechained protocol and the blockchain platform, e.g., no Merkle trees, blocks have no nonce, a simplified calculation of Rechained’s proofID calculation. Further limitations originate from the limited scripting capabilities of CPN-Tools, e.g., the implemented hashing function does not provide real hashing properties. Similar applies to the symbolic implementation of public-key cryptographym which only allows for the symbolic signing of hashed data records.

Because Rechained is a cryptocurrency-based protocol, it suffers from volatile cryptocurrency prices that complicate its everyday use.

Finally, Rechained may allow network operators to determine the cost of a Sybil node attack, but it neither fully prevents such an attack, nor does it help to actually detect the Sybil nodes. However, depending on the scenario, Blockchain analysis techniques [76] could be employed to help identify identities created by the same entity.

## 7. Conclusions and Future Work

Once Sybil nodes have entered a network, detecting them can be an error-prone and cumbersome process. In this work, we propose Rechained, which introduces a direct and adjustable per-identity cost and disincentivizes Sybil attacks, even before they start. Our protocol is fully decentralized and uses public blockchains to offload the proof-of-work process, while, at the same time, providing offline verification and identity revocation mechanisms. Rechained is formally evaluated by means of Colored Petri Nets, includes revocations and support for SSI.

Our analysis shows that circumventing the security mechanisms of Rechained incurs equivalent or higher costs for an attacker than following the protocol as intended, showing the suitability of the scheme for disincentivizing the creation of spurious or malicious identities.

Ino order to create new identities, which can even be used in scenarios where eclipse attacks have to be considered, Rechained binds blockchain-based wallet address (i.e. cryptographic public and private key pairs) to identities. During this process, an identity proof is generated, which can be verified offline. The identity proof further contains an unpredictable proof ID. Identity creation has an adjustable price tag and different types of payment (i.e., to a network operator, to charity, or proof of burn) are described. When connecting to a network secured by Rechained, nodes exchange identity proofs and verify them before proceeding to connect. The generation of separate revocation proofs is also supported.

Cryptocurrencies are somewhat notorious for their highly volatile prices, but our analysis shows that, even in the worst case, this volatility impacts the security of Rechained only to a very limited degree. Network parameter update mechanisms allow further adjustment in case of price or difficulty changes. For networks with at least intermittent Internet connectivity, we describe different ways of adjusting network parameters to accomodate a changing environment. At the same time, we detail approaches that can be used to keep the network’s configuration in working order, even in the complete absence of Internet connectivity, which makes Rechained suitable for many different operating environments.

We also formalize the protocol by means of Colored Petri Nets to analyze its properties and behavior and provide a basic proof-of-concept implementation that shows the suitability of Rechained for low powered nodes, such as those that may be found in Internet of Things applications.

In the future, we plan to investigate the feasibility of implementing our scheme on blockchains based on other consensus mechanisms than PoW, as it is often criticized for its high energy consumption. We also plan to evaluate further IoT use-cases for Rechained in the real world. Finally, employing blockchain analysis techniques should be investigated, as it could be a promising approach for identifying possible attacks that occur, despite the economic disincentivization.

## Figures and Tables

**Table 1:** Overview of Rechained parameters.

Parameter	Description
minHeight	Identity proofs have to refer to blocks of at least this height on the chain.
receiver	One or multiple addresses that receive the payment for transaction creation.
amount	The minimum amount of crypto currency that has to be sent to create an identity.
amountLocked	Optional: A timelocked output sent back to the user, with the lock time determining the lifetime of the identity.
minDifficulty	The minimum mining difficulty required for an identity proof to be valid.

**Table 2:** Behavioral interfaces of activities for Rechained—extension of [5].

Activity	Trigger	Pre-Condition	Post-Condition
Create Deposit Transaction	User wants to create a deposit transaction	Network parameters, machine identity and machine wallet	Network parameter, machine identity, machine wallet, deposit transaction
Mining	Received deposit/revocation transaction	Deposit/revocation transaction, previous block hash, deposit/revocation wallet and blockchain difficulty target	Block, previous block hash, blockchain difficulty target, deposit/revocation wallet
Create Identity Proof	Deposit transaction mined into block and user wants to create new identity proof	Block with deposit transaction, machine identity and machine wallet	Identity proof, machine identity, machine wallet
Validate Identity Proof	Incoming identity proof	Identity proof, network parameter, machine identity and machine wallet	Boolean statement whether the provided identity proof is valid, or not
Create Revocation Transaction	User wants to create a revocation transaction	Identity proof, network parameter, machine identity and machine wallet	Network parameter, machine identity, machine wallet, revocation transaction and identity proof
Revoke Identity Proof	Revocation transaction mined into block	Block with revocation transaction, identity proof, identity revocation list, machine identity and machine wallet	Identity proof, machine identity, machine wallet and identity revocation list

**Table 3:** Exemplary acronyms, names, and description of token colors of the Rechained CPN model—extension of [5].

Token Color	Description	Type
KeyPair	Key pair	(pubKey, privKey)
Wallet	Blockchain wallet	(Address, Balance)
NetworkParameter, NP	Rechained network parameter	(Difficulty, minBlockHeight, minDeposit, depositAddress)
Difficulty	Minimum PoW difficulty for an identity proof as defined by the network operator	Integer
minBlockHeight	Minimum block height as defined by the network operator	Integer
minDeposit	Minimum deposit to be made for an identity proof as defined by the network operator	Integer
depositAddress	Deposit address as defined by the network operator	String
Transaction, TX	Structure of a deposit transaction	(ID, from, to, amount, pubKey, txSig)
Block	Blockchain block	(ID, prevBlockHash, BlockchainDiffTarget, txList)
IdentityProof, IP	Identity proof	(BlockID, BlockHash, BlockchainDiffTarget, TXID, BlockTXList, proofID, proofSig)
proofID	proofID as specified by the protocol	String
MachineIdentity	Machine entity identity	(DID, KeyPair)
depositWallet	Deposit wallet as defined by the network operator	Wallet
machineWallet	Machine’s wallet	Wallet
IdentityRevocation-List, IRL	List of IDs of revoked identity proofs	[Integer]
validationResult	Result of the identity proof validation	Boolean

**Table 4:** Affected starting dates after which the Bitcoin price drops below a certain percentage of the given day’s price between January 2017 and April 2021.

Drop to	Affected Start Dates
<10%	0.0%
<20%	0.8%
<30%	4.6%
<40%	10.1%
<50%	22.0%
<60%	36.3%
<70%	43.2%
<80%	52.6%
<90%	60.0%
<100%	81.1%

**Table 5:** Affected starting dates after which the Ethereum price drops below a certain percentage of the given day’s price between January 2017 and April 2021.

Drop to	Affected Start Dates
<10%	2.5%
<20%	14.8%
<30%	25.9%
<40%	31.7%
<50%	40.6%
<60%	45.8%
<70%	54.3%
<80%	60.3%
<90%	71.5%
<100%	89.3%

**Table 6:** State-space analysis results of the Rechained CPN model.

Loops	Home Markings	Dead Markings	Dead Transitions	Live Transitions
No	No	Yes	No	No

## Data Availability

The data presented in this work are openly available in IEEE DataPort at http://dx.doi.org/10.21227/e5tk-3y77.
